# Disseminated tuberculosis following the placement of ureteral stents: a case repot

**DOI:** 10.1186/1757-1626-1-383

**Published:** 2008-12-10

**Authors:** Bashar Salem

**Affiliations:** 1 Hospitalist/Internal Medicine Department, Hillcrest Medical Center, 1145 South Utica Ave, Suite 1105, Tulsa, OK 74104, USA

## Abstract

**Background:**

Miliary tuberculosis occurs as a result of hematogenous dissemination of Mycobacerium tuberculosis. This can occur due to progressive primary infection, reactivation of latent focus with subsequent spread, or rarely via iatrogenic origin.

**Case presentation:**

This is a case of 21 year-old woman presented with hydronephrosis and hematuria due to unrecognized renal tuberculosis. She underwent bilateral ureteral stent placement which lead to dissemination of the Mycobateria through the blood causing pulmonary tuberculosis and multiple tuberculous abscesses in the paraspinous muscles, pleural space and skin.

**Conclusion:**

Disseminated tuberculosis due to Mycobacteremia after surgical intervention is a rare complication. Mycobacteria should be considered among other more common microorganisms that can cause post operative bacteremia.

## Background

Miliary tuberculosis accounts for 1–2% of all cases of tuberculosis and about 8% of all forms of extrapulmonary tuberculosis in immunocompetent individuals. It's more frequent in immunosuppressed patients. [1]

The lymphohaematogenous dissemination of M. tuberculosis is crucial step in the development of miliary tuberculosis. This can occur during early generalization from primary disease or during late generalization from reactivation of latent focus which can be pulmonary or extrapulmonary.

## Case presentation

This is a 21 year-old Native American woman, without known medical disease or immunodeficiency. She had recurrent dysuria for about four months. She was treated with multiple courses of antibiotics for urinary tract infections. Two months later, she developed hematuria and abdominal CT was performed and showed bilateral hydronephrosis with unspecific etiology. She was admitted to a local hospital and underwent bilateral ureteral stents placement and discharged on antibiotics. Three weeks later, she was transferred to our facility complaining of severe back pain, fever and flank pain. Her previous surgical history is significant for right salpingectomy after tubal pregnancy two years ago. On physical exam, she had high temperature 102°F. The right costovertebral angle was tender. There were two nontender skin nodules on the lateral side of the right thigh and posterior calf.

Her laboratory data showed: White blood cell count 13,200; Hemoglobin 11.5; Platelets 340,000; Erythrocyte sedimentation rate 103; Serum Sodium 133; Serum Creatinine 0.5; Albumin 2.8, liver enzymes were normal. Urine analysis showed WBC > 100, RBC > 100, and protein 1+. Routine urine culture and blood cultures had no growth. HIV test was negative. TB skin test was nonreactive.

**Figure 1:**
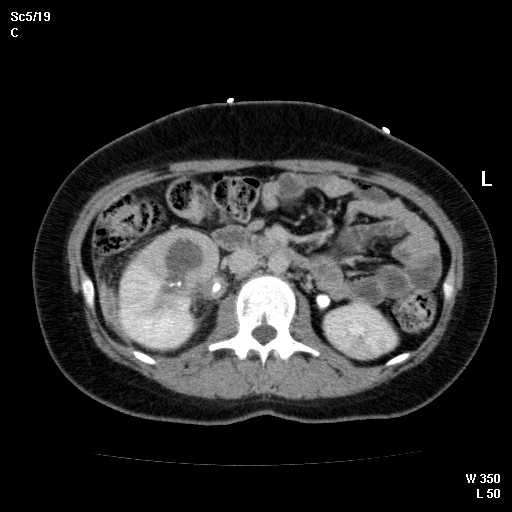
Computerized tomography of the abdomen showing 3.8 cm abscess in the anterior cortex of the right kidney.

**Figure 2:**
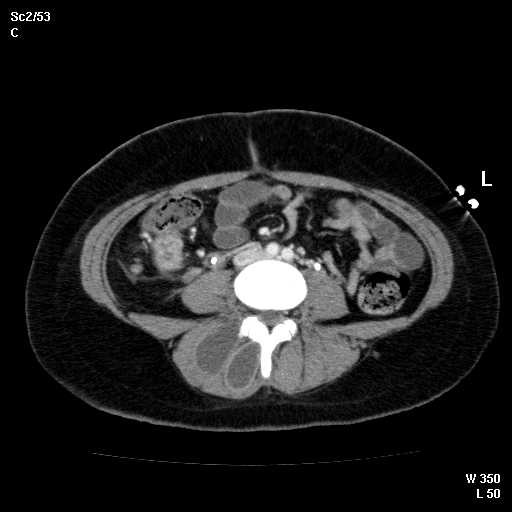
Computerized tomography of the pelvis showing rim enhancing abscess in the right paraspinous muscles with early extension into the vertebra.

**Figure 3:**
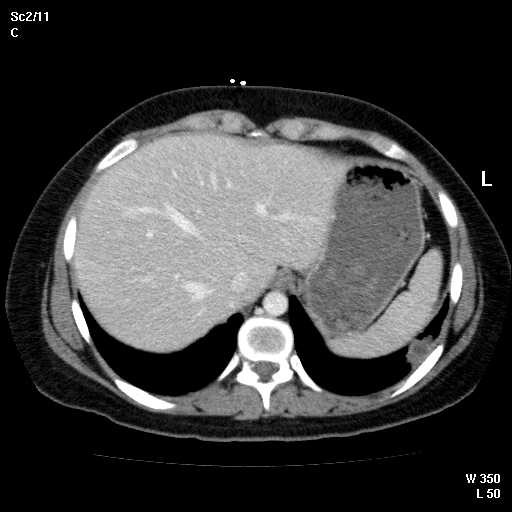
Pleural – based small abscess with low density center in the lateral aspect of left lower lobe of the lung.

**Figure 4:**
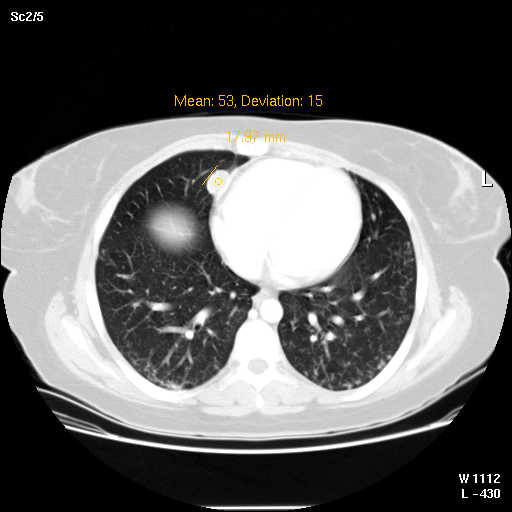
1.8 cm lesion attached to the right pericardium.

Abdominal CT showed new 3.8 cm low density lesion in the anterior cortex of the right kidney without hydronephrosis (Figure 1), right paraspinous muscle abscess extending from L2 to S3 with some bony destruction (Figure 2), left gluteus medius muscle abscess, and multiple small lesions in the pleural space (Figure 3), one of them is attached to the pericardium (Figure 4). Chest x-ray showed diffuse nodular infiltrates.

She underwent CT guided needle aspiration of the paraspinal abscess. The smear for acid fast bacilli was negative, but AFB culture grew Mycobacterium *tuberculosis *on day 16. Meanwhile, in the setting of this metastatic pattern, patient underwent multiple diagnostic tests. A punch skin biopsy of the cutaneous nodules showed multiple areas of fat necrosis with noncaseating granulomas, negative for AFB staining. A video-assisted thoracoscopic lung biopsy showed caseating granulomas with many acid fast bacilli. Miliary tuberculosis was confirmed on day 14. Later on, Mycobacterium tuberculosis was isolated from many specimens: urine, skin tissue, muscle abscess, bronchoalveolar lavage and the lung tissue. It was sensitive to all antimycobacterial agents. Patient was started on 4 drug regimen. She had CT – guided placement of a drainage catheter into the paraspinous muscle abscess, then was removed after one week. She had her ureteral stents exchanged. She was afebrile upon discharge on day 28 and continued to improve.

## Discussion

The patient had initially local urinary symptoms for four months consistent with renal tuberculosis, which is a common form of extrapulmonary tuberculosis. It remains dormant for many years after the kidneys become seeded during the primary tuberculous infection. It is often an insidious disease presents with local symptoms including dysuria, hematuria, sterile pyuria, flank pain. The constitutional symptoms such as fever, weight loss, and night sweats are uncommon. They were found only in 14% in one series by simon et al [2]. Absence of symptoms was up to 20% in the same study. Indeed, the diagnosis sometimes will be made for the first time at post mortem. The disease is usually found in both kidneys in pathological studies even if it presented in one side clinically. The kidney function is preserved unless the destruction is advance and bilateral. The radiologic abnormalities include parenchymal necrosis, calcification, ureteric strictures, calyceal dilatation with distortion, renal cavitations, and bladder fibrosis. Urine acid fast bacilli cultures are positive in 80 – 90% of genitourinary tuberculosis and it is essential in making the diagnosis.

Our patient didn't have fever or any symptoms to suggest the involvement of any organ other than the kidneys until she had the ureteral stents placed. Her first CT scan of the abdomen showed hydronephrosis only. There were no abscesses in the kidney or the paraspinous muscles. Few days after the procedure she began to develop fever, back pain, skin lesions in the right leg. Three weeks later, a repeat CT scan showed multiple abscesses and dissemination of the mycobacteria. The placement of the ureteral stents has most likely introduced the mycobacteria into the blood stream causing pulmonary tuberculosis, muscular, paraspinal, cutanous and subpleural tuberculous abscesses. Miliary tuberculosis can occur due to iatrogenic causes [1]. This is one of few cases described in the literature where miliary tuberculosis occurred after instrumentation of the renal sytem such as ureteral catheterization [3], retrograde pylogram [4], extracorporeal shockwave lithotripsy [5] or laser lithotripsy [1]. The miliary disease was reported after surgical intervention in a tuberculous epididymitis [6], intraocular tuberculosis [7]. Intravesical BCG immunotherapy for transitional cell cancer of the bladder [8], intralesional BCG injection for melanoma and osteogenic sarcoma treatment have all lead to miliary tuberculosis in several repoted cases. Treatment with anti-TNF alpha monoclonal antibodies and corticosteroids increase the risk of opportunistic infections including tuberculosis. Transplant with unrecognized tuberculosis – infected cadaveric kidney [9] or homograft valve [10] were also reported.

Surgical intervention along with chemotherapy is useful in patients who require decompression and stabilization of the spinal cord, abscess drainage, and/or debridement of infected material. In some circumstances, reconstructive surgery of the spine, joints, ureter or any affected area may be needed after completion of the antituberculosis regimen. Interventions after starting antituberculosis chemotherapy in diagnosed patients are safer and should not cause complicated mycobacteremia. Ureteral stenting in renal tuberculosis, combined with medications usually decrease the likelihood of the renal loss, but may increase the opportunity for later reconstructive surgery. [11]

## Conclusion

Renal tuberculosis is an indolent disease. It needs high level of clinical suspicion, especially when sterile pyuria is encountered. Mycobacteremia after surgical intervention is a rare complication. It usually occurs when the tuberculous infection goes unrecognized preoperatively. Mycobacteria should be considered among other more common microorganisms that can cause post operative bacteremia.

## Consent

Written informed consent was obtained from the patient for publication of this case report and accompanying images. A copy of the written consent is available for review by the Editor-in-Chief of this journal.

## Competing interests

The author declares that they have no competing interests.
